# Association between frailty status and health literacy in the elderly

**DOI:** 10.4314/ahs.v25i1.37

**Published:** 2025-03

**Authors:** Jingli Kou, Qiuping Li, Yanqiu Wang, Min Yue, Shenshen Yang

**Affiliations:** 1 Department of Geriatrics, Xuanwu Hospital of Capital Medical University, Beijing 100053, China; 2 Department of Nursing, Xuanwu Hospital of Capital Medical University, Beijing 100053, China

**Keywords:** elderly, frailty, health literacy

## Abstract

**Background:**

To analyze the association between frailty status and health literacy in the elderly, and identify the influencing factors of health literacy the elderly. Provide a basis for enhancing the health literacy of elderly patients.

**Methodology:**

Relevant data, including general information, frailty status and health literacy level, were collected using the convenience sampling method from 185 elderly people attending inpatient or outpatient clinics in a Grade-III Class-A hospital in Beijing.

**Results:**

Among the elderly people, the prevalence of frailty was 23.2%, and the overall health literacy was at a high level, with a rate of health literacy possession of 84.9%. Results of analysis of variance and trend test revealed a linear relationship between frailty status and health literacy, i.e., health literacy level of the elderly became lower as the degree of frailty increased. It was found by multivariate linear regression analysis that the educational level, frailty status, daily activity and type of medical insurance were independent influencing factors for of health literacy (P<0.05).

**Conclusion:**

The health literacy level varies among the elderly with different frailty status,. Health literacy and frailty has s dose-response relationship. and t Frailty is an independent influencing factor of health literacy. Targeted education can be given based on different characteristics of the elderly to raise their health literacy level.

## Introduction

Frailty is a pathophysiological state of rapid decline in multiple physiological functions of the body associated with increasing age, which can cause homeostasis disorders, leading to incapacity, readmission and even death^1^,^2^. With increasing population aging, the prevalence of frailty is on the rise^3^, and it was 18.7%, 20.6%, and 28.4% in 2011, 2013, and 2015, respectively, among the elderly in China. Health literacy refers to the ability of individuals to access, understand and process basic health information and services, and use these resources to make decisions conducive to improving self-health^4^. The “Healthy China 2030” promulgated in China clearly states the need to strengthen health education ad requires that the national health literacy level should reach 30% by 2030^5^. However, elderly people are a disadvantaged group in terms of health literacy, and they have lower health literacy levels than young people^6^. A low health literacy level is strongly associated with morbidity and mortality in the elderly^7^,^8^. The elderly with frailty have worse overall body function, which may have a greater impact on their health literacy. The above findings emphasize the importance and need for concern about health literacy in older frailty patients within the context of a rapidly aging population. Most previous studies in this area have investigated the associations between health literacy and frailty, but whether frailty is an independent factor of health literacy is unclear. In this study, the association between frailty and health literacy in the elderly was analyzed, and the impact factors of health literacy were analyzed by multivariate linear regression analysis. It can provide references for further research and improvement of health literacy in the elderly.

## Patients and methods

### Patients

Elderly people attending inpatient or outpatient clinics in a Grade-III Class-A hospital in Beijing from February to March 2022 were selected as subjects using the convenience sampling method. Inclusion criteria were set as follows: 1) elderly people aged ≥65 years, 2) those who had clear consciousness and were able to cooperate in this survey, and 3) those who voluntarily participated. Exclusion criteria involved elderly people with severe mental disorders or severe cognitive dysfunction.

### Survey tools

#### General information questionnaire

Information on the age, gender, educational level, type of medical insurance, type of chronic diseases, economic conditions and residential status of the subjects was collected. The chronic diseases mainly included hypertension, diabetes mellitus, cerebral-cardiovascular diseases, coronary heart disease, and chronic nephritis.

#### Health literacy

The health literacy of the elderly was assessed using the Health Literacy Scale for Patients with Chronic Diseases. Converted into Chinese edition by Sun HL et al^9^ in 2012 based on the Health Literacy Management Scale developed by Professor Jordan in Australia, the scale consists of 24 items in 4 dimensions, including 9 items on ability to obtain information, 9 items on competence to communicate and interact with others, 4 items on willingness to improve health, and 2 items on willingness to support financially. Using the 5-point Likert method, “completely difficult”, “very difficult”, “somewhat difficult”, “not very difficult” and “not at all difficult” are scored 1-5 points, the total score is 0-120 points, and the higher the score, the higher the health literacy level of the patient. According to the Analysis Guide of the First Health Literacy Survey Report of Chinese Residents, the subject was considered to have health literacy if he or she could correctly answer 80% or more of the questions on health literacy^10^. As shown in the testing of validity and reliability among 430 patients with chronic diseases, the total Cronbach's α coefficient of the scale is 0.894, the Cronbach's a coefficient of each dimension is 0.857-0.940, and the retest reliability is 0.683^11^.

#### Frailty status

The frailty status was assessed using the FRAIL Scale proposed by experts from the International Task Force on Nutrition and Aging in 2008. The scale consists of 5 indicators assessed by 5 self-assessment questions: (1) Do you feel frail? (2) Can you climb a flight of stairs by yourself without a break? (3) Can you walk 500 meters by yourself without a break? (4) Do you suffer from more than 5 diseases? (5) Have you lost more than 5% of your body weight in the last year? The total score is 0-5 points, and ≥3 points, 1-2 points and 0 points are considered frailty, pre-frailty and no frailty, respectively. With good reliability and validity (evidence grade III, recommendation grade B)^12^, the scale has been used in several countries^13^.

### Data collection

A questionnaire survey was conducted by three trained investigators. The principle of anonymity and confidentiality was followed, and the subjects were informed of the purpose and methods of this study before the survey. The survey was conducted only after the patients voluntarily signed the informed consent form. In a face-to-face interview, questions were raised by the investigator in the same words. Then the filled questionnaires were recycled on site, checked and verified item by item, and the questionnaires that had missing items or were not standardized were re-filled promptly. A total of 200 questionnaires were issued, 187 were recycled, and 2 invalid questionnaires were excluded, with an effective recovery rate of 92.5%.

### Statistical analysis

Data were entered using EpiData 3.1, and Statistical Product and Service Solutions (SPSS) 24.0 software (IBM, Armonk, NY, USA) was used for statistical analysis. Continuous variables were described by mean ± standard deviation or median and interquartile range, categorical variables were expressed by frequency and constituent ratio, and the Mann-Whitney U test or Kruskal-Wallis H rank sum test was used for comparison between groups. The differences in health literacy were compared among the elderly with different frailty status by analysis of variance, and the linear relationship between frailty and health literacy was analyzed by trend test. Multivariate linear regression analysis was conducted (αin=0.05, αout=0.1). P<0.05 was considered statistically significant.

## Results

### General information of the elderly

A total of 185 elderly people were enrolled, including 113 (61.1%) males and 72 (38.9%) females. The average age was (77.3±8.14) years old, and the average score of activity of daily living was (90.27±13.79) points. They suffered from 2 (1,3) diseases on average. In terms of the educational level, there were 63 (30.1%) cases of junior high school or below, 47 (25.4%) cases of senior high school/technical secondary school, and 75 (40.5%) cases of junior college or above, showing an overall high educational level. Medical insurance for urban residents was dominant (84 cases, 45.4%). Family economic conditions were considered good in 39 (21.1%) cases, not bad in 69 (37.3%) cases, moderate in 72 (38.9%) cases and poor in only 5 (2.7%) cases. As to living status, they mostly lived with others (162 cases, 87.6%) including their spouse and children.

### Frailty status of the elderly

Frailty occurred in 43 (23.2%) cases, pre-frailty in 72 (38.9%) cases and no frailty in 70 (37.9%) cases. The incidence of frailty had differences among the elderly with different types of medical insurance, family economic conditions, scores of activity of daily living, and number of comorbidities. Specifically, the elderly with employee medcal insurance had a higher prevalence rate of frailty.

The elderly with frailty had poorer family economic conditions, poorer activity of daily living, and more comorbidities than those with pre-frailty or no frailty.

### Health literacy level of the elderly

The health literacy score of the elderly was (106.76±12.76) points, and the rate of health literacy possession was at a high level (84.9%). To be specific, the scores of ability to obtain information, competence to communicate and interact with others, willingness to improve health, and willingness to support financially were (40.35±5.4) points, (38.38±6.37) points, (18.34±2.41) points and (9.69±0.88) points, respectively (Table 2).

**Table 2:** Health literacy level of the elderly (n=185)

Item	Score range (point)	Score (χ̅ ± s)	Health literacy possession (%)
Ability to obtain information	9-45	40.35 ± 5.4	151 (81.6)
Competence to communicate and interact with others	9-45	38.38 ± 6.37	137 (74.1)
Willingness to improve health	4-20	18.34 ± 2.41	165 (89.2)
Willingness to support financially	2-10	9.69 ± 0.88	180 (97.3)
Total score	24-120	106.76 ± 12.76	157 (84.9)

### Association between frailty status and health literacy in the elderly

The health literacy level was compared among the elderly with different frailty status. The results showed that the total score of health literacy and the score of each dimension except for the willingness to support financially had significant differences among the elderly with frailty, pre-frailty and no frailty. The trend test is a statistical method specifically designed to deal with ordered categorical data. We used it to identify if frailty, an ordered data, has a dose-response relationship with health literacy. It shows that the literacy became worse as the degree of frailty increased. Meanwhile, ability to obtain information, competence to communicate and interact with others and willingness to improve health also became worse as the degree of frailty increased (Table 3). It means frailty and literacy have a dose-response relationship.

**Table 3:** Trend test between health literacy and frailty of the elderly (n=185)

Item	No frailty	Pre-frailty	Frailty	*χ* ^2^	*p*
Total score of health literacy	109.60±9.91	107.31±10.65	101.23±17.74	17.727	<0.001
Ability to obtain information	41.53±4.21	40.68±4.26	37.86±7.86	13.741	<0.001
Competence to communicate and interact with others	39.57±5.44	38.32±6.36	36.56±7.41	18.432	0.015
Willingness to improve health	18.79±1.86	18.57±1.89	17.23±3.48	3.365	0.067
Willingness to support financially	9.71±0.75	9.74±0.87	9.58±1.07	3.907	0.048

### Multivariate linear regression analysis of health literacy in the elderly

In order to identify the influencing factors of health literacy of the elderly people, the correlation analysis was carried out firstly to find which general variances were correlated to health literacy. According to Table 4, it revealed that gender, educational level, type of medical insurance, activity of daily living, outpatient/inpatient, economic condition, and frailty status were related to the elderly health literacy. Multivariate linear regression analysis was conducted with health literacy as the dependent variable and the related variables carried out before of the elderly as the independent variables. Meanwhile, continuous variables were incorporated with original values, categorical variables were assigned, and dummy variables were set as unordered categorical variables (Table 5). It was found that the educational level, frailty status, daily activity and type of medical insurance were independent influencing factors for health literacy. In other words, a higher educational level corresponded to a higher health literacy score. Frailty status was negatively correlated with the health literacy score, and the patients with frailty had a lower health literacy score. The health literacy score was higher among the elderly with employee medical insurance and public medical care than that among the elderly with medical insurance for urban residents (Table 6).

**Table 4:** Correlation analysis of health literacy and participants' general information (n=185)

Item	n	Health literacy	*r*	*P*
Type of patients			-0.216	0.003
Outpatient	56	110.05±11.58		
Inpatient	129	105.33±13.02		
Age (Y)			-0.086	0.245
65-75	77	107.75±14.25		
76-85	74	105.74±14.25		
>85	34	106.74±10.19		
Gender			-0.131	0.075
male	113	108.13±12.07		
female	72	104.61±13.59		
Educational level			0.338	<0.000
Junior high school or below	63	101.52±14.33		
Senior high school/technical secondary school	47	107.15±10.20		
Junior college or above	75	110.92±11.27		
Type of medical insurance			0.097	0.191
Medical insurance for urban residents	84	105.87±13.84		
Employee medical insurance	52	106.81±10.52		
New rural cooperative medical system	12	94.33±19.82		
public medical care	37	112.76±4.85		
Family economic condition			-0.187	0.011
Poor	39	109.62±9.82		
Moderate	32	107.16±13.93		
Not bad	69	106.08±11.14		
Good	50	88.80±2.41		
Living status			0.004	0.955
Solitary	23	108.17±9.29		
Non-solitary	162	106.56±13.19		
Activity of daily living			0.352	<0.000
100	78	111.37±8.75		
61-99	95	103.53±14.27		
<60	12	102.42±13.68		
Number of comorbidities (n)			0.007	0.929
0-1	69	106.93±12.43		
2-4	83	106.84±10.90		
>4	33	106.21±17.40		
Frailty			-0.276	<0.000
No frailty	41	111.22±8.99		
Pre-frailty	119	107.32±10.49		
Frailty	25	96.80±20.83		

**Table 5:** Independent variable assignment

Variable	Assignment
Gender	Male =1, female =2
Educational level	Junior high school or below =1, senior high school/technical secondary school =2, junior college or above =3
Type of medical insurance	Medical insurance for urban residents (0, 0, 0), employee medical insurance (1, 0, 0), new rural cooperative medical system (0, 1, 0), public medical care (0, 0, 1)
Living status	Solitary =1, non-solitary =2
Economic condition	Poor =1, moderate =2, not bad =3, good =4
Outpatient/inpatient	Outpatient =1, inpatient =2
Frailty status	No frailty =1, pre-frailty =2, frailty =3
Daily activity	100=1, 61-99=2, <60=3

**Table 6:** Multivariate linear regression results of health literacy of the elderly (n=185)

Independent variable	Regression coefficient	Standard error	Standardized regression coefficient	*t*	*p*
Constant	88.968	9.759	-	9.117	< 0.001
Educational level	2.089	0.769	0.221	2.716	0.007
Frailty status	-4.315	1.563	-0.200	-2.761	0.006
Daily activity	3.678	1.720	0.173	2.138	0.035
Type of medical insurance	1.057	0.613	0.125	1.723	0.087

Note: R2=0.206, adjusted R2=0.174, F=6.546, p<0.001

## Discussion

### The frailty status of the elderly is of concern

The prevalence of frailty in this study was 23.2%, slightly lower than that of elderly inpatients investigated by Sun et al.^14^ but significantly higher than that (7.4-14.2%) of the community-dwelling elderly in China. There is a lack of a consensus on frailty assessment, and different tools are applied in different studies on frailty assessment, which may have some influence on the results of frailty screening. However, as confirmed in previous literature, the prevalence of frailty is generally higher in inpatients than that in the community-dwelling elderly^16^,^17^. In this study, the elderly people attending inpatient or outpatient clinics were enrolled, and these people were more prone to weakened systemic reserve function and reduced anti-stress ability due to diseases, so they suffered from frailty. The frailty status of the inpatient and outpatient elderly deserves more attention from medical workers, and early screening and intervention should be conducted to avoid adverse prognostic events. Besides, frailty occurred more easily in the elderly with poorer family economic conditions, poorer activity of daily living and more comorbidities, consistnt with the literature report. In a survey involving 1,400 elderly people over 60 years of age, Yang et al.^17^ found that low monthly income and incapacity are influencing factors for frailty. Han et al.^18^ argued that the prevalence of frailty is higher in elderly patients with severer comorbidities and poorer activity of daily living. Unlike previous studies^19^, the difference was not statistically significant in the correlation between age and frailty in this study. However, the trend of study data showed that the prevalence of frailty increased with age, and the negative results may be attributed to the small sample size in this study.

### The health literacy level is higher among the elderly

The health literacy score was (106.76±12.76) points, and the rate of health literacy possession was 84.9% in this study, higher than that (69.2%) in the study by Sang et al.^20^, which was mainly related to the characteristics of the elderly enrolled. Most of the elderly inpatients in this study came from the Cadre Healthcare Ward, and the elderly outpatients were mainly from the Cadre Healthcare Clinic and Special Need Clinic. These people possess better social conditions and resources, and can access higher levels of medical resources to support health management. In addition, up to 97.3% of these people had a willingness to support financially, suggesting better economic conditions that can also help them obtain appropriate health knowledge and resources to the greatest extent^21^. Meanwhile, the elderly with an educational level of senior high school or above accounted for 62.5% of the elderly in this study, showing an overall higher level. Comparatively, the elderly with more academic knowledge and higher decision-making ability can better identify false inormation and more actively seek health support. The health literacy level in this study is also higher than that reported by National Health Commission of the People's Republic of China in 2021^21^. This may be due to differences in populations and assessment tools. Among the four dimensions of health literacy, the best literacy is willingness to support financially, and the worse dimension is competence to communicate and interact with others. It is of good value for our clinical work. It indicates that the elderly is eager to obtain health protection and intervention skills, but they lack the skills to communicate with others. We clinical workers need to focus on target dimension to help the elderly to promote health.

### Frailty has a dose-response relationship with health literacy

Frailty is associated with high risk of several common adverse outcomes in the elderly. The results revealed that frailty has a dose-response relationship with health literacy. Health literacy has been demonstrated to have a significant role in promoting self-care in patients^22^. The finding can serve as points of reference for the design of care strategies for elderly people. We clinical workers need to pay more attention on the promotion of health literacy especially the frailty elderly. The elderly who has not suffer from frailty experienced less mobility restriction and consequently, better self-care ability. Health literacy has been demonstrated to high level of positive information seeking, attitude change, and behavioral change, and increase motivation and autonomy in the context of adherence to frailty management strategies^23^. A better physical condition led to a better health literacy. The results indicate the effective assessments and intervention strategies for frailty is important for elderly people to promote health mentally.

### The influencing factors of health literacy of the elderly

The results of this study revealed that the health literacy level differed among the elderly with different frailty status, and except for the willingness to support financially, the overall health literacy, ability to obtain information, competence to communicate and interact with others and willingness to improve health became worse as the degree of frailty increased, which was also verified by the results of multivariate linear regression analysis. Frailty indicates the poorer health status of the elderly, whereas the elderly without frailty are relatively healthy, because they may be more concerned about health literacy and development of a healthy lifestyle^24^.

Moreover, health literacy is associated with non-adherence^25^, i.e., the lower the health literacy of patients, the higher the non-adherence to pharmacological and behavioral interventions. In this study, patients with frailty had lower health literacy, and they might behave poorly in individual frailty management, thus further worsening frailty. As a result, a vicious circle was formed between low health literacy and frailty, which may greatly increase the risk of adverse outcomes in the elderly. Therefore, the target elderly population should be focused on, and appropriate approaches should be adopted to improve their health literacy level according to their knowledge level, experience, frailty status and other characteristics, thereby further raising their quality of life. In this study, the results of multivariate linear regression analysis also showed that a higher educational level corresponded to a higher health literacy score, and the health literacy score was higher among the elderly with employee medical insurance and public medical care than that among the elderly with medical insurance for urban residents, the former of whom had higher abilities to screen, recognize, analyze and comprehend the health information. The government of China is also making great efforts to carry out health care reform to provide well-established medical security for its people. The result indicated that elderly people values health enough to be willing to support on finance. A more secure form of insurance can be a good support to fetch health information and skills. Education level was significantly related to health literacy. A better educational level allows them more chance to master the skills to acquire health knowledge and information to form a high-level self-management efficacy. They tend to maintain physical exercise and reasonable diet to improve the quality of life and rehabilitation effect in many aspects. As the same to frailty, a high level of daily activity maintains the physical ability to obtain a high-level health literacy. Previous studies suggested that health literacy was positively correlated to self-management efficacy and quality of life^26^. Limited health literacy is a serious public health problem^27^. It is related to adverse outcomes. The health literacy should be a health priority in clinical work place.

## Conclusion

This study revealed the dose-response relationship between health literacy and frailty in older adults in Peking. The results showed that the elderly with frailty had lower levels of health literacy than those without frailty, and frailty was a significant influencing factor for health literacy in the elderly, providing a new controllable factor for subsequent studies on health literacy improvement in the elderly. Meanwhile, the improvement of health literacy of the elderly can reverse the frailty process. In future clinical practice, therefore, the health literacy level and treatment compliance of the elderly can be improved by taking into account the frailty status and paying attention to the method of health information dissemination and education.

## Limitations

Our study had some limitations. Firstly, Tthe subjects participates enrolled were mainly retired cadres, so might lead to a low generalizability. Secondly, the sample was small that might lead to a sample bias. Thirdly, the variables evaluated using a cross-sectional design; therefore, further prospective studies are needed to evaluate the effect of health literacy on the elderly health conditions. In the future, more studies related to health literacy in large samples and different characteristics of the population are required.

## Figures and Tables

**Table 1:** General information of the elderly (n=185)

Item	No frailty [n (%)]	Pre-frailty [n (%)]	Frailty [n(%)]	z	p
Type of patients				-1.743	0.081
Outpatient	24 (42.9)	25 (44.6)	7 (12.5)		
inpatient	46 (35.7)	47 (36.4)	36(27.9)		
Age (Y)				N/A	0.291
65-75	34 (44.2)	28 (36.4)	15 (19.4)		
76-85	25 (33.8)	31 (41.9)	18 (24.3)		
>85	11 (32.4)	13 (38.2)	10 (29.4)		
Gender				-1.299	0.194
Male	47 (41.6)	42 (37.2)	24 (21.2)		
Female	23 (31.9)	30 (41.7)	19 (26.4)		
Educational level				N/A	0.154
Junior high school or below	19 (30.2)	25 (39.6)	19 (30.2)		
Senior high school/technical secondary school	18 (38.3)	19 (40.4)	10 (21.3)		
Junior college or above	33 (44.0)	28 (37.3)	14(18.7)		
Type of medical insurance				N/A	<0.001
Medical insurance for urban residents	45 (53.6)	33 (39.3)	6 (7.1)		
Employee medical insurance	10 (19.2)	18 (34.6)	24 (46.2)		
New rural cooperative medical system	4 (33.3)	5 (41.7)	3 (25.0)		
Public medical care	11 (29.7)	16 (43.2)	10 (27.1)		
Family economic condition				N/A	<0.001
Poor	0 (0.0)	1 (20.0)	4 (80.0)		
Moderate	19 (26.4)	24 (33.3)	29 (40.3)		
Not bad	28 (40.6)	32(46.4)	9 (13.0)		
Good	23 (59.0)	15 (38.5)	1 (2.5)		
Living status				-1.021	0.307
Solitary	6 (26.1)	11 (47.8)	6 (26.1)		
Non-solitary	64 (39.5)	61 (37.6)	37 (22.9)		
Activity of daily living				N/A	<0.001
100	41 (52.6)	32 (41.0)	5 (6.4)		
61-99	29 (30.5)	37(38.9)	29 (30.5)		
<60	0 (0.0)	3 (25.0)	9 (75.0)		
Number of comorbidities (n)				N/A	<0.001
0-1	39 (56.5)	27(39.1)	3 (4.3)		
2-4	31 (37.3)	32(38.6)	20 (24.1)		
>4	0 (0.0)	13(30.239.4)	230 (60.669.8)		
health literacy	109.60±9.91	107.31±10.65	101.23±17.74	6.163	0.003

**Note:** N/A: not applicable, no statistical values shown in inter-group comparison using Kruskal-Wallis H rank sum test
